# Supplementary material for: Potential for Drug Repositioning of Midazolam as an Inhibitor of Inflammatory Bone Resorption
Source: Int J Mol Sci. 2024 Jul 12;25(14):7651. doi: 10.3390/ijms25147651 (PMC11277201; doi:10.3390/ijms25147651)
Supplement: Supplementary file 1 [file ijms-25-07651-s001.zip › ijms-3075294-supplementary.pdf]

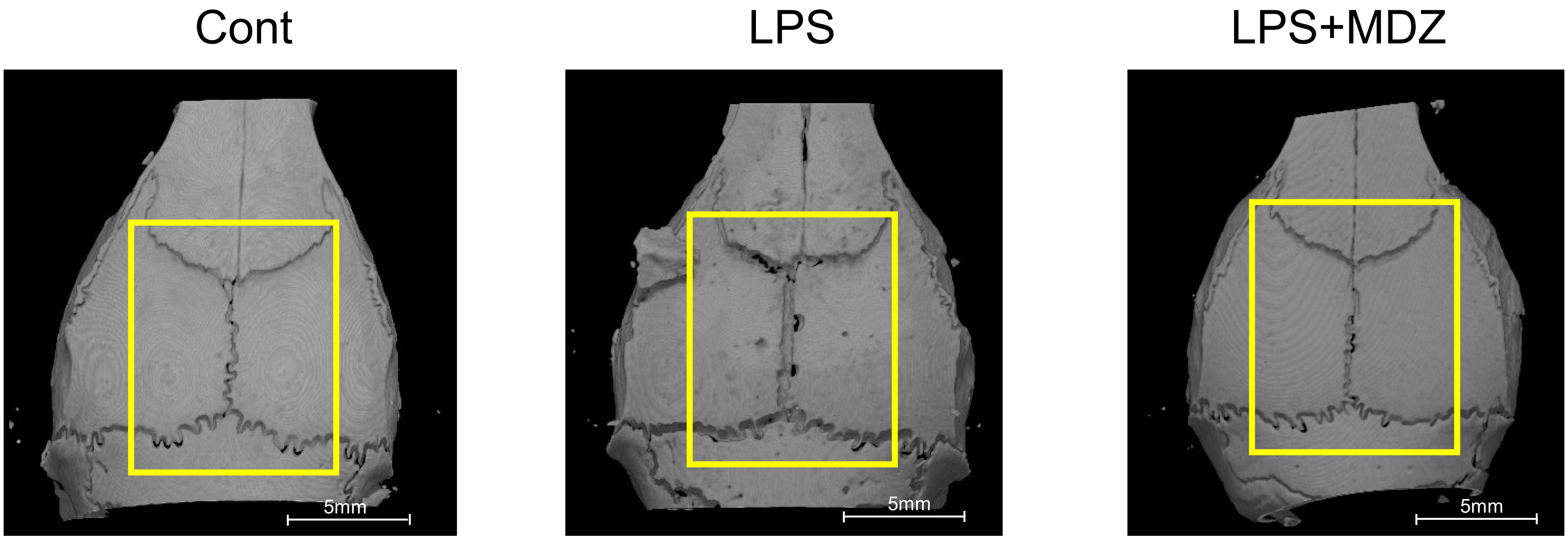

Figure S1. Original images of micro-CT 3D reconstruction images of mouse calvarias shown in the main Figure 4B. The cropped parts that are shown in the main Figure 4B are marked by yellow boxes. Cont: PBS-alone dosage group (i.e., control); LPS: LPS-only dosage group; LPS+MDZ: combination of LPS and MDZ dosage group. LPS: lipopolysaccharide; MDZ: midazolam. Scale bar = 5 mm.

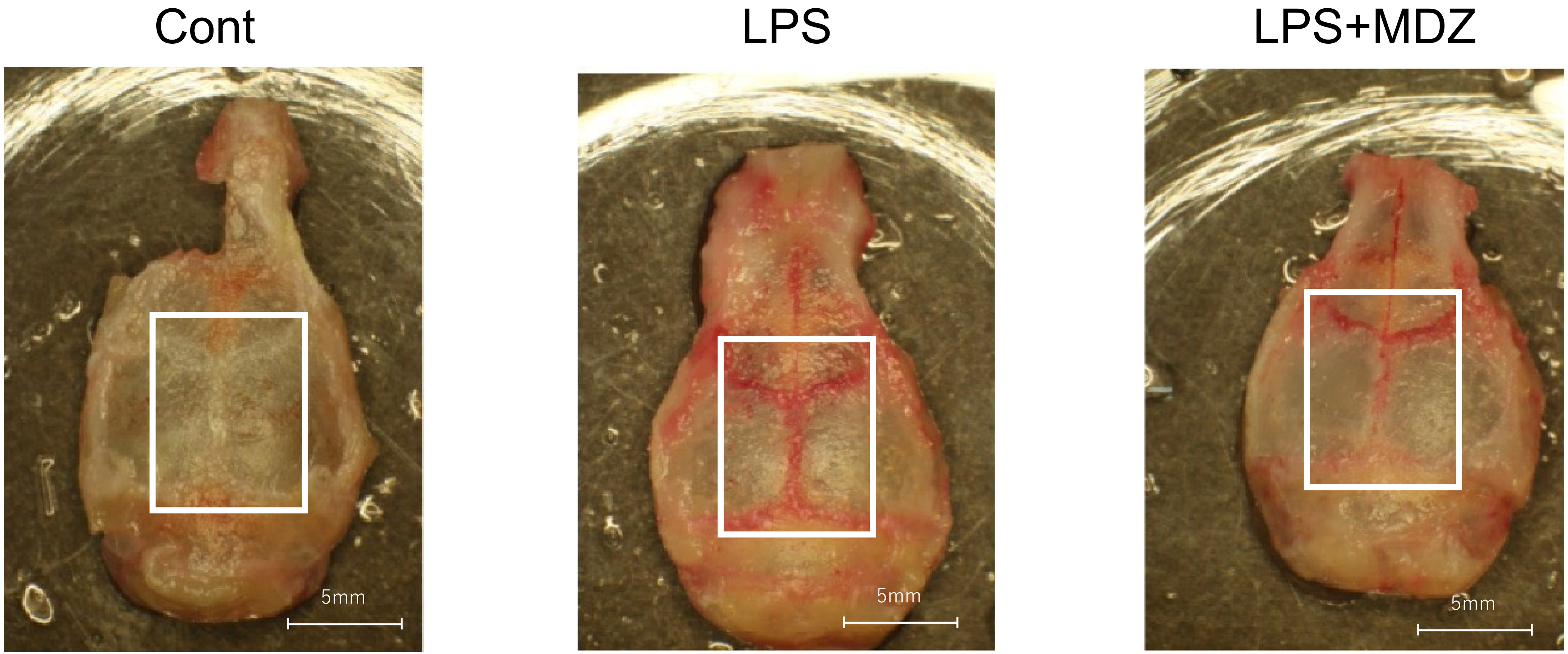

Figure S2. Original images of stereomicroscopic images of TRAP staining shown in the main Figure 4C. The cropped parts that are shown in the main Figure 4C are marked by white boxes. Cont: PBS-alone dosage group (i.e., control); LPS: LPS-only dosage group; LPS+MDZ: combination of LPS and MDZ dosage group. LPS: lipopolysaccharide; MDZ: midazolam; TRAP: tartrate-resistant acid phosphatase. Scale bar = 5 mm.

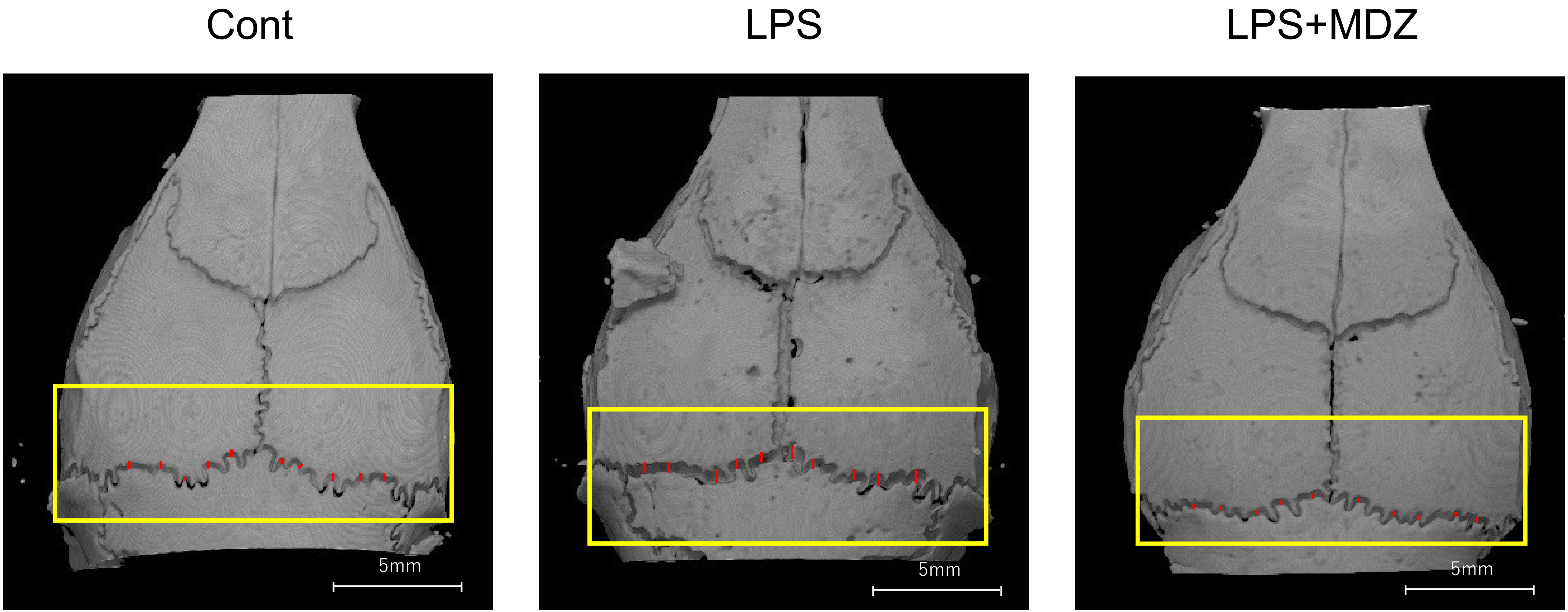

Figure S3. Original images of micro-CT 3D reconstruction images of mouse calvarias shown in the main Figure 5A. The cropped parts that are shown in the main Figure 5A are marked by yellow boxes. Cont: PBS-alone dosage group (i.e., control); LPS: LPS-only dosage group; LPS+MDZ: combination of LPS and MDZ dosage group. LPS: lipopolysaccharide; MDZ: midazolam. Scale bar = 5 mm.

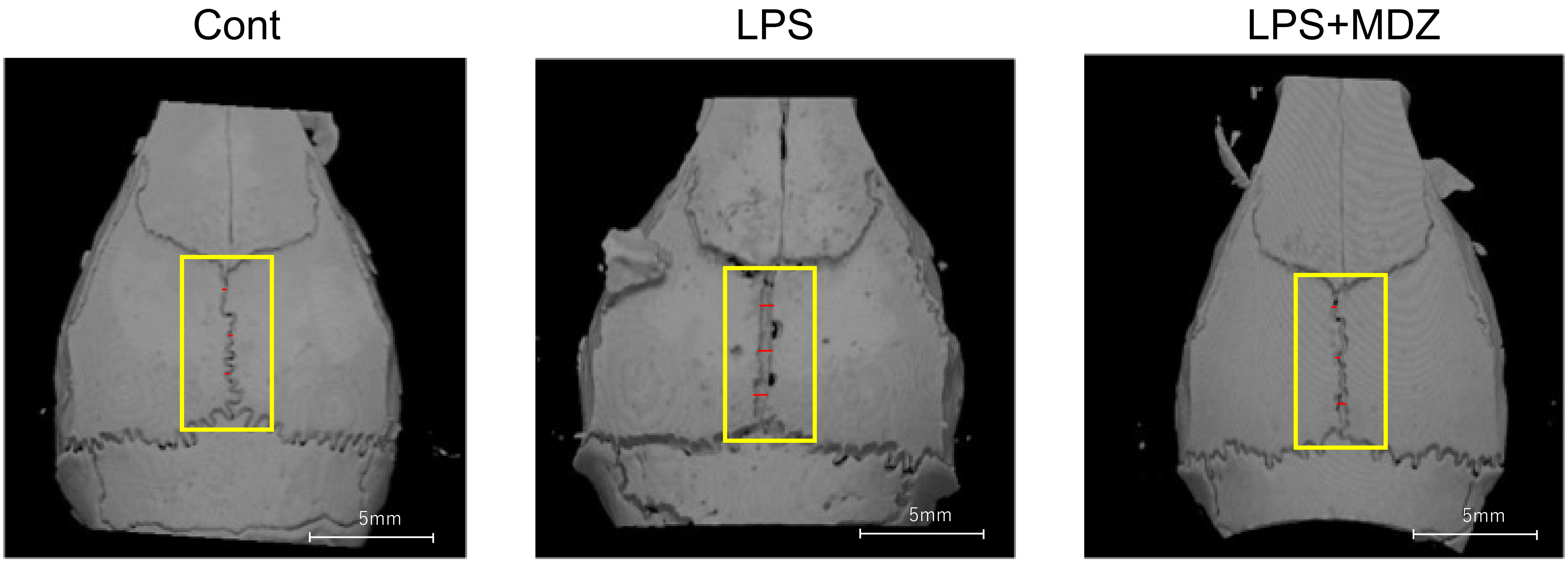

Figure S4. Original images of micro-CT 3D reconstruction images of mouse calvarias shown in the main Figure 5B. The cropped parts that are shown in the main Figure 5B are marked by yellow boxes. Cont: PBS-alone dosage group (i.e., control); LPS: LPS-only dosage group; LPS+MDZ: combination of LPS and MDZ dosage group. LPS: lipopolysaccharide; MDZ: midazolam. Scale bar = 5 mm.

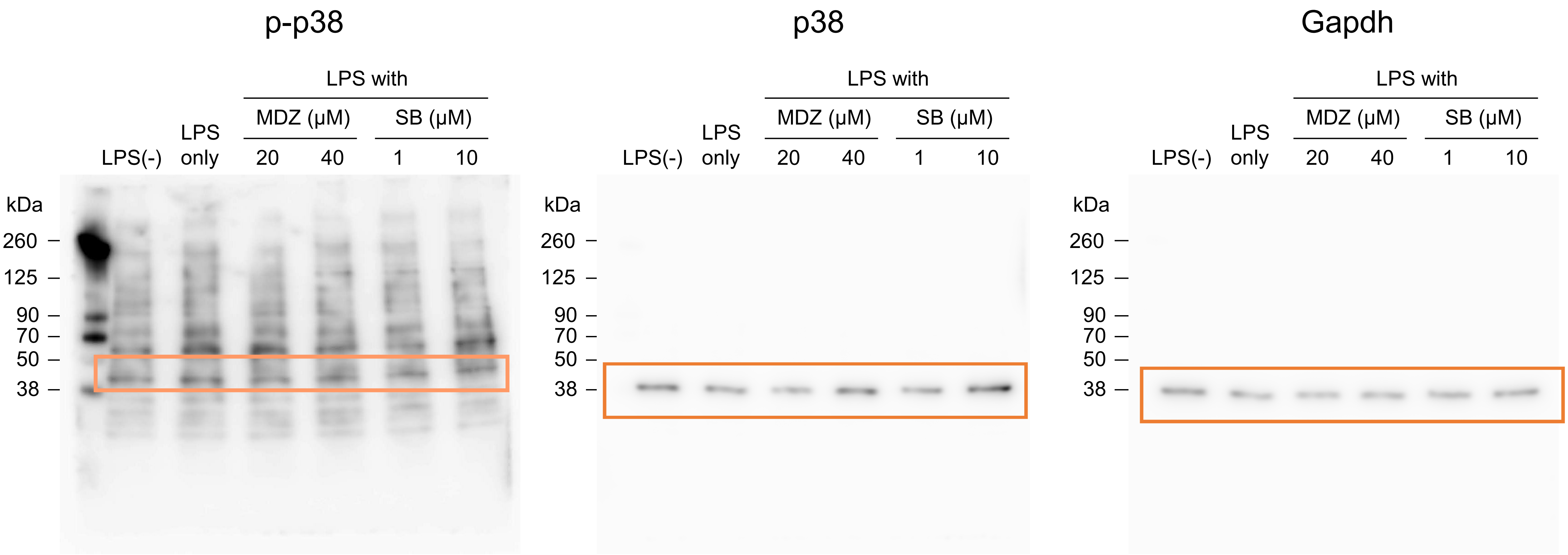

Figure S5. Uncropped images of all Western blots shown in the main Figure 7B. The cropped parts that are shown in the main Figure 7B are marked by orange boxes. LPS: lipopolysaccharide; MDZ: midazolam; SB: SB203580. LPS(-): RAW264 cells cultured without LPS; LPS only: RAW264 cells cultured with LPS alone. p38: p38 mitogen-activated protein kinase (p38 MAPK), p-p38: phosphorylated p38 MAPK, Gapdh: glyceraldehyde-3-phosphate dehydrogenase.

Table S1. Selected primers and size of amplified product for qPCR analysis.

Gene		Sequence (5′ --- 3′)	Product Size (bp)	qPCR Protocol (45 cycles)		
Nfatc1	F	GCCTCGAACCCTATCGAGTG	206	Denaturation	95 °C	10 sec
	R	TCCCGGTCAGTCTTTGCTTC				
Trap	F	CAGCTGTCCTGGCTCAAAA	218	Annealing	60 °C	10 sec
	R	ACATAGCCCACACCGTTCTC				
Ctsk	F	GAGGGCCAACTCAAGAAGAA	203	Extension	72 °C	15 sec
	R	GCCGTGGCGTTATACATACA				
Gapdh	F	CCATCACCATCTTCCAGGAG	346			
	R	ACAGTCTTCTGGGTGGCAGT				

F: forward, and R: reverse
